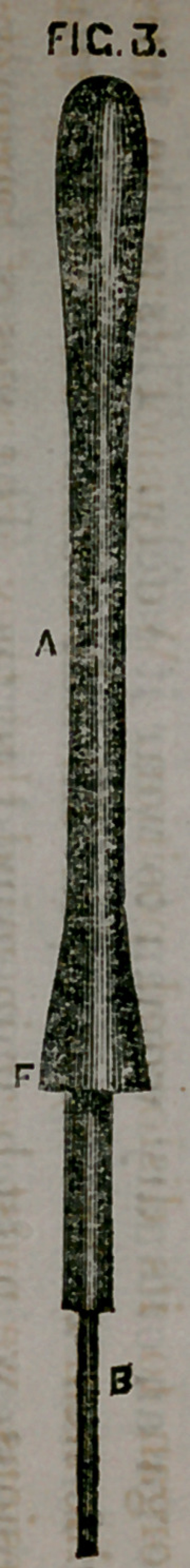# Treatment of Retro-Flexions of the Uterus

**Published:** 1874-11

**Authors:** Ely Van DeWarker

**Affiliations:** Syracuse, New York


### TREATMENT OF RETRO-FLEXIONS OF THE UTERUS.

BY ELY VAN DEWARKER, M.D., OF SYRACUSE, N. Y.

In the October number, for 1873, of the *New York Medical Journal*, I first called the attention of the profession to a new form of self-retaining intra-uterine stem. A cut of the stem was included in the article. In the *Medical Record* for December 15, 1873, the peculiarities of the treatment of uterine flexions by that form of stem was given with an illustration of the instrument. In the *Buffalo Medical and Surgical Journal* for April, 1874, a longer article appeared with details of four cases. This paper contained a cut of a form of intra-uterine stem, the devising of which anteda- ted that of the self-retaining form. This stem—an illustration of which accompanies the text of this paper—is a modification of that of Dr. Graily Hewitt.

The articles referred to treatment of flexions in general. But subsequent use of the vaginal flange intra-uterine stem in the man- agement of retro-flexions convinced me that it was a very valuable means, not alone of cure, but what is oftentimes equally important, of relief from present pain.

I also ascertained that the self-retaining form of stem was pecu- liarly adapted to the correction of ante-flexions, being retained better, and worn with more comfort than any other form of intra- uterine support. I am induced, therefore, in this paper, to divide the subject, and confine my remarks to the treatment of the sim- pler form of flexion,—that of retro-flexion—and in some future paper, when I have had command of a larger field of observation, to treat of the subject of anti-flexion.

Those who have had their attention called specially to the sub- ject of woman's diseases, I think will coroborate me when I say that a flexion is the greatest form of uterine luxation. I cannot help regarding the assertion of Mr. Bernutz that flexions of the womb are without symptoms, and "unaccompanied by any functional disturbance," to be altogether foreign to the truth. Mr. Burnutz gets his start in this theory from M. Paul Debois, and has only the credit of carrying the theory to an extreme never intended by Baron Dubois. (*Diseases of Women; Burnutz and Goupil, New Syndenham Society. Vol. II., p.* 193.) I have a case in my mind now—case 4 of the table—in which a marked form of retro-flex- ion exists without any evidence of functional impairment, and this after a year or more of great suffering. I am positive that in this case her former symptoms were due to the flexion, and I am equally sure that her present freedom from pain is simply a measure of the habitude of the system at large, and of the organ itself to the new situation. I believe that the womb may, and often does, accommo- date itself to a change of axis or of curve, and this after periods of great suffering; just as the head of the femor will make for itself a new resting place after a dislocation, and repose in its abnormal position without pain or distress after a due interval.* I have observed yet another case in which I detected an acute state of backward flexion, the only evidence of uterine derangement being a catarrh of the cervix. Menstrually, her health was perfect. It is neeeless to say I did not treat this flexion. I can sum up my experience of the efforts of uterine flexions thus, briefly: That I have been astonished at the amount of nervous disturbance and pain which may result from the flexion of the womb, as a rule. The science of gynæcology in reference to uterine flexions is undergoing a complete revolution. The old fear of intra-uterine splints for the correction of this deformity is giving away before the demonstrated fact that a support of this character may be constructed so that it may be worn not only safely, but with comfort. In designing the first form of my instrument, I adopted the shelf form of Dr. Hewitt. Everything about his instrument was too large. I reduced the size of the stem at least one-half. I next reduced the enormous vaginal collar to one-fourth or one-fifth the size, and yet had my instrument perfectly retained. From Dr. Hewitt's description and figures of his instrument, it seems impossible for an ordinary woman to wear it safely. This is the reason that former writers have expressed their condemnation of the intra-uterine stem in such unmeasured terms. The stems formerly in use have violated anatomy, physiology, and mechanics alike. Theoretically, it should be of just sufficient size to afford grasp to the vagina to retain both stem and flange, or whatever else the vaginal portion may consist of, in position, and yet accommodate the changing positions of the womb. The error in the construction of stems hitherto in use, seems to have existed in this fact: that the inventors had a very vague idea of how fixed a body in the grasp of the vagina may be. I found that the flange could be made five-eights to an inch in diameter, and when of this size could be worn and perfectly retained for months. That I had, in a measure, answered the theoretical requirements, was shown by the fact

*In the splendid museum of the Albany Medical College, now Medical Department of Union University, there are several specimens of old dislocations of the thigh, in which the bead of the bone had formed functionally almost perfect joints in the new situation.

that its pressure afforded exemption from pain, and, of itself, never became a source of irritation. Its mode of operation is simple. The stem *a*, fig. 3, upon the end of the wire *b*, being in position, is introduced into the cavity of the womb, being used like an ordinary sound without a speculum; the flange *a*, fig. 2, by means of its central perforation *c*, is placed upon the wire. Two slots *e*, upon opposite sides of the perforation *c*, receive the wire, so that one being pushed up, the flange presents itself at the ostium vagina by its edge, enters the parts without difficulty, and is gently forced up the wire until it rests against the collar of the stem. The instrument is then in position, and the wire is withdrawn.

FIG. 2

FIG. 3

Whether we use a stem or not in the treatment of a flexion, we must be governed by the circumstances of the case. In the first place, it is, of course, understood that there exists no lurking perimetric or parametric inflammation, either acute or chronic. It is a matter of common experience how easily a slumbering cellular or intra-pelvic peritoneal trouble can be roused into troublesome if not dangerous activity by untimely manipulation. It may even exist potentially in the tissues, and surprise us by springing suddenly into life. If this condition is discovered, it must be treated, and no matter how long a time may have elapsed, it must be removed.

The next thing to be considered, is the womb in such a condition as to bear the persistent pressure of a foreign body in its cavity. In some conditions of the uterus and of the nervous system, the slightest intrusion of a foreign body into its cavity, leads to serious results. I bear in mind one case in which convulsions and insensibility resulted from the introduction of a sound.

Some idea of the amount of uterine tolerance may be formed by the tenderness with which the organ responds to the touch. In a retro-flexion, for instance, when the finger comes in contact with the posterior uterine wall in the retro-vaginal *cul de sac*, a keen sensibility to even slight pressure may be elicited. The introduction of a sound or repositor would in such a case probably cause great pain. There are two ways of treating this. The first is by frequently passing a sound and leaving it in position for ten or twenty minutes, and repeating this from day to day until the sensitiveness of the lining membrane of the uterus is changed. A quicker, and I think better way, is to treat the inordinate sensibility of the endometrium by the free use of the intra-caustic instrument. This is sometimes followed by acute pain, which I think is better treated by a medicated pessary, charged with morphia, than by that drug either by the mouth or hypodermically. It may be necessary to repeat this three or four times before the acute sensibility is removed.

Another point to be considered is the adhesions which may bind the organ to its dislocated position. We cannot trust an intra-uterine stem to break, or relax old adhesions. In treating these adhesions, we must bear in mind that they are traces of parametric inflammation ; that they may not be traces alone, but inflammation may be lurking potentially in the tissues of the parts, and rough or untimely handling may rouse it into dangerous activity. If the adhesions are firm, it is evident that the fundus could not ascend from its depressed position, and if the stem were introduced, the body of the uterus could only be straightened by throwing the neck violently forward and upward, thus placing the organ at nearly right angles to the axis of the vagina. In a case some time since under treatment, I could not throw the uterus into position by the repositor, on account of the great pain resulting from such attempts; but at the expense of much time, I succeeded in forcing the fundus upward and forward with a pair of sponge probangs, after the manner of Dr. Sims in retroversion. By this manipulation, often repeated, the fundus yielded more and more from its retro-flexed position, by either the breaking up or the elongation of the fibres of the adventitious tissue. If the adhesions resist this treatment, I cannot suggest any means of overcoming them. A case in this unfortunate condition would, I think, be best treated by a tamping of carbolized cotton-wool, which would give a considerable degree of support to the organ.

One other point in the treatment of retro-flexions, and I finish. A stem will correct a flexion, but is powerless to overcome a version. I am speaking of intra-uterine stems with small vaginal attachments. Thus, my vaginal flange stem would correct a version to the extent of just one-half the diameter of the flange. So far as the uterine supports are implicated, a flexion is an intensified form of version; the sustaining forces of the fundus being more involved in the former than in the latter state of dislocation. Whatever version may be present after the introduction of a stem —if it requires any treatment at all—must be treated independently of the stem. The most efficient means of overcoming the version, I have found, consists of a tamping of cotton saturated with carbolized glycerine in the posterior vaginal *cul de sac*. It must be just bulky enough to offer a firm bed to the body of the organ, but not so large that it will protrude beyond the neck, otherwise it will be brought within the grasp of the vagina and expelled together with the stem. I have removed such a tamping after it had been in position for ten days, and found it free from any unpleasant odor. It ought to be changed once a week.

Dr. Routh, Dr. Chambers, and other English writers speak of dilating the os with sponge tents, or dividing the cervix by hysterotome, in order to introduce a stem (*Trans. of the Obstetrical Society of London*, XV., 252). This may be necessary in order to introduce the finger into the uterine cavity; but it seems to me to be entirely unnecessary to the introduction of a proper stem. A stem should be so made that it will enter wherever a sound will pass. As a rule, the os in either form of flexion is patulous, and readily permits the introduction of the stem.

With this paper is a tabulation of thirteen cases of retro-flexion treated with the stem. Five other more recent cases are now under observation, in which the stem is well borne, and giving relief, but they are of too recent occurrence to include in the table.

Messrs. Sheperd & Dudley, 150 William street, New York, will make the stem to the order of those who wish to test it.

*Table of Cases of Retro-flexions of the Uterus Treated with the Vaginal Flange Intra-uterine Stem.*

| No. | HISTORY. | NATURE OF MECHANICAL SUPPORT. | SUBSIDIARY TREATMENT. | LENGTH OF TIME UNDER OBSERVATION. | RESULTS. |
|---|---|---|---|---|---|
| 1 | Age 43; seamstress; multipara and abortions; uterine hyperplesia. | Vaginal flange stem | Seton in cervix tonics, saline lax. | 2 years, stem worn 8 months. | Flexions has disappeared; a tendency to version existing; entire relief of symptoms; general health good. |
| 2 | Age 28; housekeeper; multipara. | Self-retaining and vaginal flange stem | Tonics, cotton tamping. | 15 months. | Discharged cured; after about 3 months returned, with retro-flexion partially existing; stem re-introduced; well borne, with entire relief of symptoms. |
| 3 | Age 43; domestic work; 12 children; intense suffering from pain and nervous symptoms; in bed for several months. | Vaginal flange stem | Tonics, pot. brom., cotton tamping. | 10 months. | Cured; in ten days after introduction of stem was about her household duties. |
| 4 | Age 44; multipara and abortions; great pain and nervous symptoms. | Self-retaining stem. | | One office visit. | Stem removed by her physician: could not be borne; is now in good health; flexion incurred. |
| 5 | Age 28; unipara; total disability. | Vaginal flange stem | | 5 months. | Cured. |
| 6 | Age 35: multipara; impairment of locomotion; general health and nervous system. | Vaginal flange stem | Cotton tamping. | 8 months. | Great relief of symptoms; stem worn 4 months continuously. |
| 7 | Age 30; multipara; very nearly total disability. | Vaginal flange stem | | 4 months. | Stem well borne, and with great comfort. |
| 8 | Age 29; menstrual unipara; derangement, and general impairment of strength. | Vaginal flange stem | Cotton tamping. | 6 months. | Stem retained three months at a time; great relief. |
| 9 | Age 36; unipara; frequent urination; constipation; pain in walking; nervous derangement | Vaginal flange stem cotton tamping. | Saline lax. and bitter tonics. | 5 months. | Stem worn with great comfort, and attending to her daily duties. |
| 10 | Age 40; widow; multipara; general health good; pain in defecation, and inability to remain long upon the feet. | Vaginal flange stem | Saline lax. | 2 months. | Walks much easier; stem has not been removed. |
| 11 | Age 30; never married; abortion two years ago; has suffered one year; great pain in sexual intercourse and in right side. | Vaginal flange stem | | 5 months. | She removes the stem herself, and frequently calls to have it replaced on account of relief it affords. |
| 12 | Age 32; no occupation; miscarriage two years ago; nervous and gastric disturbance; great pain in walking. | Vaginal flange stem cotton tamping. | Pepsin, pot. brom. | 7 months. | Had great terror of the stem; said it caused great pain in right side; stem removed, and treated solely with cotton tamping; relief of symptoms. |
| 13 | Age 40; multipara; symptoms principally menstrual. | Vaginal flange stem | | 11 months. | Stem worn three months continuously; has had no treatment since; says she is better. |

## Figures and Tables

**FIG. 2. f1:**
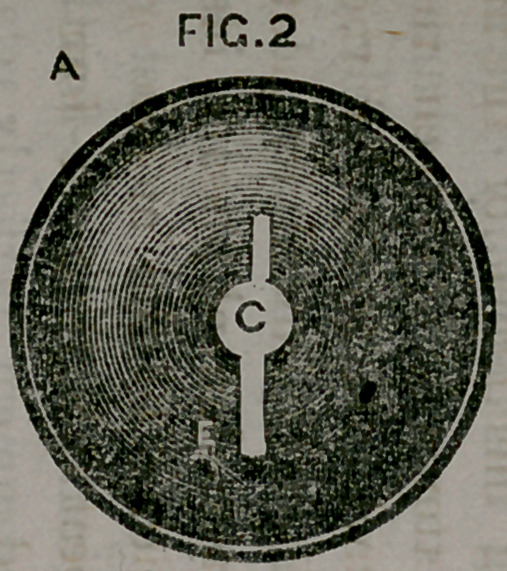


**FIG. 3. f2:**